# Classification and Assessment of the Patelar Reflex Response through Biomechanical Measures

**DOI:** 10.1155/2019/1614963

**Published:** 2019-07-09

**Authors:** Yolocuauhtli Salazar-Muñoz, G. Angelina López-Pérez, Blanca E. García-Caballero, Refugio Muñoz-Rios, Luis A. Ruano-Calderón, Leonardo Trujillo

**Affiliations:** ^1^Tecnológico Nacional de México/Instituto Tecnológico de Durango, C.P. 34080, Durango, DGO, Mexico; ^2^Universidad Politécnica de Durango, C.P. 34300, Durango, DGO, Mexico; ^3^Servicios de Salud del Estado de Durango, Hospital General 450, C.P. 34206, Durango, DGO, Mexico; ^4^Tecnológico Nacional de México/Instituto Tecnológico de Tijuana, C.P. 22430, Tijuana, B.C., Mexico

## Abstract

Clinical evaluation of the patellar reflex is one of the most frequent diagnostic methods used by physicians and medical specialists. However, this test is usually elicited and diagnosed manually. In this work, we develop a device specifically designed to induce the patellar reflex and measure the angle and angular velocity of the leg during the course of the reflex test. We have recorded the response of 106 volunteers with the aim of finding a recognizable pattern in the responses that can allow us to classify each reflex according to the scale of the National Institute of Neurological Disorders and Stroke (NINDS). In order to elicit the patellar reflex, a hammer is attached to a specially designed pendulum, with a controlled impact force. All volunteer test subjects sit at a specific height, performing the Jendrassik maneuver during the test, and the medical staff evaluates the response in accordance with the NINDS scale. The data acquisition system is integrated by using a tapping sensor, an inertial measurement unit, a control unit, and a graphical user interface (GUI). The GUI displays the sensor behavior in real time. The sample rate is 5 kHz, and the control unit is configured for a continuous sample mode. The measured signals are processed and filtered to reduce high-frequency noise and digitally stored. After analyzing the signals, several domain-specific features are proposed to allow us to differentiate between various NINDS groups using machine learning classifiers. The results show that it is possible to automatically classify the patellar reflex into a NINDS scale using the proposed biomechanical measurements and features.

## 1. Introduction

The observation of the patellar reflex is one of the clinical trials performed most frequently for neurological tests, making it an essential tool for the diagnosis of many neuromuscular diseases [1].

The patellar reflex is a deep tendon reflex, mediated by the spinal nerves from the levels L2, L3, and L4 in the spinal cord, predominantly in the root L4. The patellar reflex test is performed to determine the integrity of the neurological function, which is accomplished by hitting the patellar tendon below the knee cap with a test hammer [2].

The patellar reflex occurs when an abrupt change arises in muscle length; in this case, it is produced by the tendon stretching, which is caused when the hammer stroke is applied [3, 4]. The normal response must be a sudden leg extension. A reduction or exaggeration of the response is indicators of damage or interruption in the innervation of the quadriceps muscle [5].

The result of the test is commonly rated using the scales of the National Institute of Neurological Disorders and Stroke (NINDS) and the Mayo Clinic [6]; in this work, we use the former one. This scale measures the response magnitude assigning a different number of “crosses” (+), whereby zero crosses (0+) indicate an exam with no visible answer; one cross (1+) corresponds to a slight reflex; two crosses (2+) indicate a reflex in the lower half of the normal range; three crosses (3+) are a reflex in the upper half of the normal range; and four crosses (4+) mean the reflex is significantly enhanced [6].

An alteration of the patellar reflex response may be caused by several different factors, which can range from tumors in the spinal cord [7] to diseases, such as the Guillain–Barre syndrome [8] that affects the peripheral nervous system [9]. Likewise, there are other factors that can disturb the test result, such as the intensity of the stroke [10], the nervousness that the patient may experiment during the test, and the age of the patient [11].

The development of an objective quantification for the test is a goal that has arisen in recent years [10, 12–14]. Some work has attempted to quantify the test by performing motion analysis [15] in cerebral palsy children [16] and also proposed a new iPhone application to measure the reflex response [17]. Other studies have attempted to model the patellar reflex as a response from a theoretical second-order system [18].

In a previous work, this research team designed a device to measure, digitally store, and display the patellar reflex response [19], capturing the relation between velocity and the magnitude of the response [20]. The aim of this study is to analyze the captured biomechanical variables, including the angle of the knee, the velocity of the knee movement, the applied force, and the magnitude of the reflex response, in order to develop an automatic classification algorithm using digital signal processing and machine learning algorithms.

## 2. Materials and Methods

### 2.1. Setup of the Measurement System

According to the previous works of Salazar-Muñoz et al. [19, 20] and Moreno-Estrada et al. [21], the designed device uses an impact sensor as the start time marker of the test and an inertial measurement unit (IMU) to measure both the angular velocity and angular position of the leg after it receives the hammer stroke on the tendon. The measurement system consists of the following two parts.

#### 2.1.1. Mechanical Controlled Force System

The mechanical controlled force system consists of a hammer designed as a Charpy pendulum. The mechanical system consists of an aluminium pendulum rubber tip attached to a toothed gear angle with an adjustable height for the hammer initial position, which allows you to select the impact force on the patellar tendon as a function of the elevation angle of the pendulum. The tip is the same as the clinical hammer used by a physician. The physician shall place the arm in the desired position and release it manually. The force applied will be the same for all test subjects to generate their own flexion. The prototype was designed such that the elevation angle can increase from 30° to 165° in steps of 15°. In these experiments, the hammer arm was elevated to 135° and the hammer mass was 195 gr, resulting in an impact force of 0.82 N, which was validated by the Charpy pendulum equation at the mechanical engineering laboratory [21].

#### 2.1.2. Data Acquisition System (DAS)

The DAS is composed of the following elements:

- *Tapping Sensor*. The LDT0-028K piezoelectric sensor manufactured by Measurement Specialities was used, connected to a charge amplifier circuit and an instrumentation amplifier to obtain a 5 V pulse, thus detecting the instant of impact on the tendon to synchronize the other measured variables.
- *Angular Displacement and Rate Sensor*. The Sparkfun IMU number SEN-11072 was used, which has 5 degrees of freedom. It contains IDG500 2-axis gyroscope with the sensitivity set to 2 mV/°/s and ADXL335 3-axis accelerometer.
- *Control Unit*. The signals from the sensors are captured by the NI USB6009 acquisition board, using two analogue channels and a power source of 5 V for the electronic system.
- *Graphical User Interface (GUI)*. The GUI was designed in LabView to display the sensor readings in real time and save the captured signals of each test in an lvm file. Each new test generates a new file that is then imported into Matlab for later analysis. The selected sample rate is 5 kHz, and the board is configured for a continuous sample mode. The GUI shows the following indicators in real time: the angular displacement, the angular velocity, and the moment of impact on the tendon.

### 2.2. Volunteer Selection

In this work, we use a group of 106 healthy volunteers to evaluate our proposed system. All of them are students from the Faculty of Medicine at the “Universidad Juárez del Estado de Durango,” and they include both men and women. The mean age, height, and body mass for subjects were 21.5 ± 1.2 years, 1.73 ± 0.09 m, and 72 ± 13 kg, respectively. A volunteer is considered to be healthy for this study if he is not suffering from any diagnosed neurological or neuromuscular disease when the test is realized [22]. The clinical trial was carried out under the direction of the Neurology Department of the “Hospital General 450” of Durango City, Mexico. The study was approved by the Ethics and Research Committee from the hospital.

### 2.3. Measurement Procedure

Experimental tests were performed under the supervision of the physician. Two reflex tests are applied to every volunteer to develop an automatic classification algorithm using digital signal processing and machine learning algorithms. We compare the NINDS scale with the biomechanical variables registered by the designed measurement system. The volunteer must be seated in a high chair, this way his right foot never touches the floor. In order to get a high relaxation of the quadriceps muscle, the volunteer is requested to perform the Jendrassik maneuver [23]. All the tests were performed under the same conditions.

**Figure 1:**
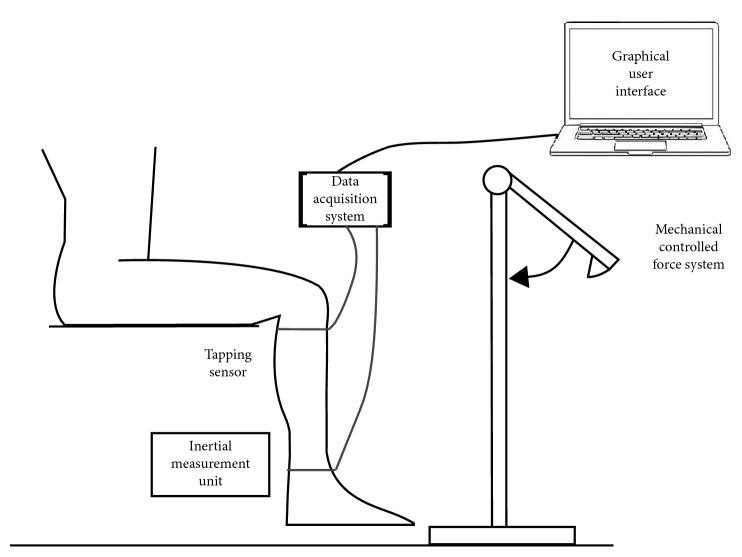
Schematic representation of the experimental system to obtain the patellar reflex response, showing the physical setup and sensor locations.

- *Test A*. A physician gives a sharp tap on the patellar tendon with a standard clinical hammer. The physician evaluates the reflex response using the NINDS scale. Dafkin et al. [10] established using stepwise multiple regression analysis that different groups of subjective raters all relied on the change of the knee angle to assess the reflex. Therefore, the trained physician was asked to focus on this feature to provide his rating for the analyzed patients.
- *Test B*. After Test A, the sensors are placed on the leg of the volunteer as shown in Figure 1, and the procedure is as follows: (a) the taping sensor (impact sensor) is adhered to the patellar tendon with tape, below the patella to avoid any undesired movements and (b) the IMU is placed on the ankle using a belt. The distance between the knee centre of rotation and location of the sensor in all subjects was maintained small following the reference [24]. The IMU must be positioned parallel to the leg and perpendicular to the floor. The controlled force system hits the patellar tendon. The data acquisition system stores all sensor readings using the GUI that was designed for this experiment. After this procedure, the measurement system is withdrawn from the leg. This test was performed under the physician who verifies that the reflex response was equivalent to Test A. No test was rejected because it ranked differently from the Test A.

### 2.4. Data Treatment and Features

The data stored by the system contain three time series. The first one is the impact signal, which marks the exact moment when the pendulum hits the tendon, denoted by *t*_o_. The second time series is the angular position signal, which measures the angle of the leg during the reflex response. The third time series is the angular velocity of the leg movement during the test. All the signals are trimmed to only extract the 4 seconds following the hammer impact, after *t*_o_, because the signal power has decreased by 97% and all the vector lengths were equal. A low pass 3^rd^ degree Chebyshev filter with a cutoff frequency of 100 Hz was used to eliminate high-frequency noise.

**Figure 2:**
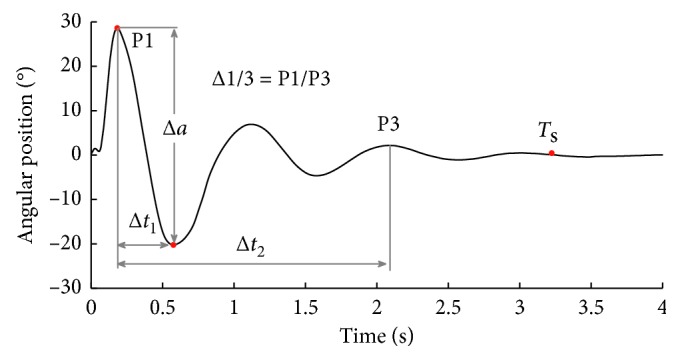
Features extracted from the angular position signal.

**Figure 3:**
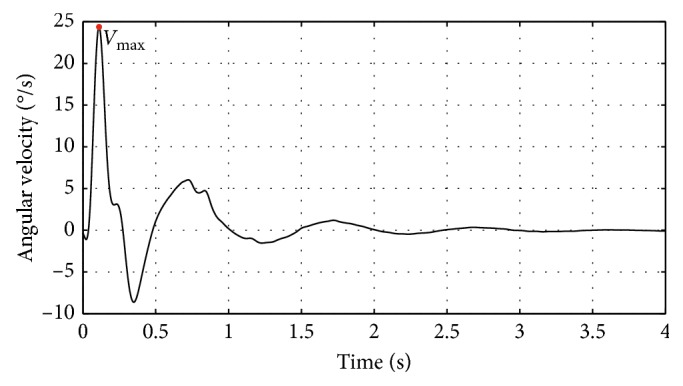
Feature extracted from the angular velocity signal.

Afterward, the signals of the angular position and angular velocity are characterized by extracting the following set of descriptive features. The extracted features are summarized in Figure 2 for the angular position and in Figure 3 for the angular velocity, each case showing a typical signal captured by the system for each measurement.

From the angular position signal, the extracted features are as follows. First, Δ*a* represents the difference between the maximum and minimum peaks of the signal. Second, Δ1/3 is the ratio between the first (P1) and third peak (P3) of the signal. Third, Δ*t*_1_ is the time interval between the maximum and the minimum peaks. Fourth, Δ*t*_2_ is the time interval between the first peak and the third peak of the signal. Finally, *T*_s_ is the settling time, which is the moment when the signal power has decreased by 97%.

In the case of the angular velocity, a single feature is extracted called *V*_max_, which is the maximum value of the signal, shown in Figure 3 as the highest peak.

### 2.5. Classification

To achieve the classification of the realized patellar reflex tests based on the number of crossings in the NINDS scale, basic pattern recognition and machine learning methods are used [25, 26]. Specifically, the following four classifiers are used:

- Naive Bayes
- Tree BAGGER
- *k*-nearest neighbors (KNN)
- Support vector machine (SVM)

Classifiers are tested with different combinations of the extracted features. Because the size of the dataset is relatively small, each classifier is tested using leave-one-out cross validation. Moreover, the data are preprocessed for feature reduction using principal component analysis (PCA).

## 3. Results and Discussion

According to the assessment given by the hospital staff at the “Hospital General 450,” the collected samples are distributed in the NINDS scale as follows: 8 samples belong to the 0+ level, 20 samples were from 1+ level, 48 samples from 2+ level, and 30 samples belong to 3+ level. The 4+ level is omitted because none of the volunteers exhibited such a response.

**Figure 4:**
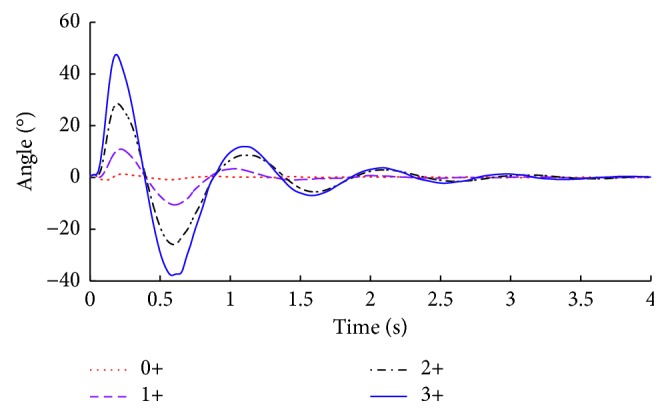
Mean signals of each NINDS group for angular position readings.

**Figure 5:**
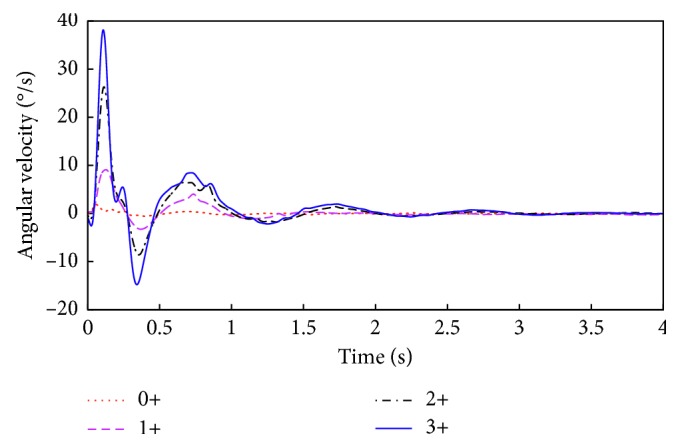
Mean signals of each NINDS group for angular velocity readings.

First, we analyze the recorded signals from each response level, to determine if there are any general similarities between them. In Figures 4 and 5, we can see the average angular position and angular velocity, grouped based on the corresponding NINDS levels.

Figure 4 shows that the movement of the leg after the impact has a wavelike behavior, which decreases with time until it stabilizes to the rest position. The maximum amplitude reached by the corresponding average signal of the 3+ group is 47 degrees. This peak corresponds to the maximum elevation of the leg. The minimum average value of the same group is −37.85 degrees, corresponding to the retraction of the leg after the lift. This value, which is the Δ*a* feature, is decreased by 36% in the corresponding average signal of the 2+ group, by 74% in the corresponding mean signal of the 1+ group, and by 97% in the corresponding mean signal of 0+ group, with respect to the mean signal of the 3+ group.

In Figure 5, the maximum value reached by the average of the velocity signals of 3+ is 38 degrees per second. This value is the *V*_max_ feature and is attenuated by 31% in the mean signal of the 2+ group, by 76% for the 1+ group, and by 95% for the 0+ group [20].

In Table 1, we can observe the mean and standard deviation of the grouped features according to the NINDS scale.

**Figure 6:**
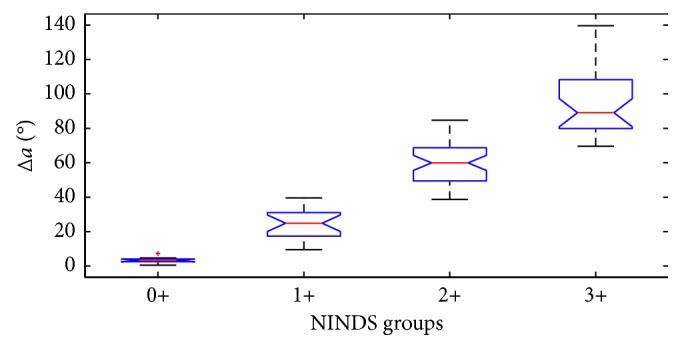
Boxplots of the Δ*a* feature for each NINDS group.

**Figure 7:**
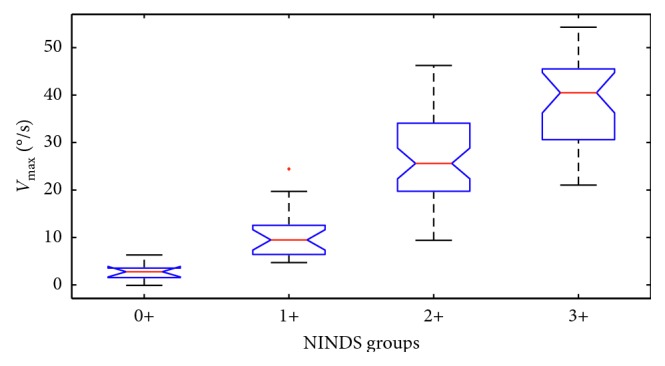
Boxplots of the *V*_max_ feature for each NINDS group.

To make sure the separation between groups is significant, the Kruskal–Wallis statistical test is applied to every feature. The test is chosen because the data distribution is not Gaussian. The test gives a *p* value <<0.05 in every test, allowing us to reject the null hypothesis that all samples share the same median.  Figure 6 shows the boxplots for each NINDS level for the Δ*a* feature, and Figure 7 shows the same box plot for *V*_max_ feature. These features are the ones that show the most separation between all the NINDS groups.

Different combinations of features are selected based on the statistical results and used as the input data for the machine learning classifiers. The tests are carried out using leave-one-out cross validation (LOO CV), given the relatively low number of samples in the database.  Table 2 shows all of the tested combinations and the classification accuracy of each classifier. In each case, principal component analysis (PCA) is applied to the input features to perform feature transformation (but results are only shown for the case in which PCA improved the performance of at least one classifier). Best performance is achieved when using the Δ*a* and *V*_max_ features with the naive Bayes classifier without PCA, with only 11 of 106 misclassifications, representing a classification accuracy of 89.62%.

**Figure 8:**
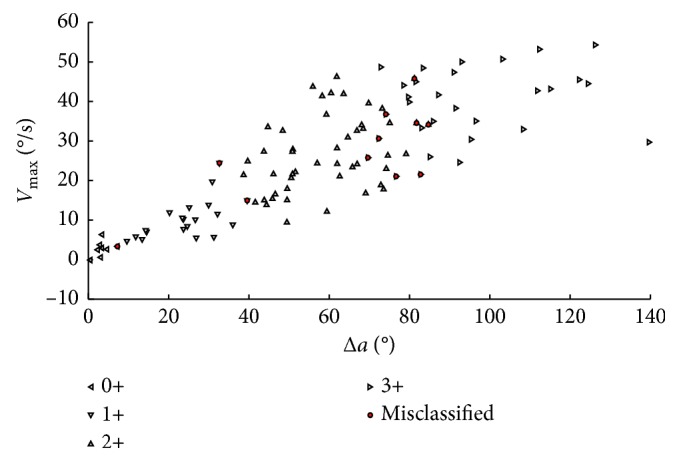
Δ*a* and *V*_max_ feature space, showing all the samples collected in the dataset. The dark round markers shows misclassified tests by naive Bayes classifier, and all other points were correctly classified into their respective groups.

Figure 8 shows all of the data samples plotted in the Δ*a* and *V*_max_ feature space. The points are labeled to show the correctly classified sample from each group, using a different mark for each NINDS level and the misclassified samples as well. Notice that most of the classification errors can be found on the boundary between the 2+ and 3+ groups.

## 4. Conclusion

The dynamic behavior of the leg during the patellar reflex creates movement patterns that can be automatically classified in the NINDS scale with a useful degree of accuracy. This is shown to be possible using a straightforward feature extraction procedure and pattern recognition techniques. The classification methods used in this study achieved a LOO CV test accuracy of 89.62% in the best case, using only two feature dimensions and the naive Bayes classifier. However, despite the good performance by the proposed system, discordance between clinical measurements and the current measurements might still be considered high in some scenarios. Moreover, the proposed approach should be verified using observations from different neurologists to determine how well this approach generalized across experts. Nonetheless, the proposed system might lead to the full automatization of this test by integrating these future improvements, along with other promising technical enhancements, such as wireless sensors to increase a patient's comfort or edge computing to simplify the data processing and transmission process.

## Figures and Tables

**Table 1:** Mean and standard deviation (mean ± std) of the features for each NINDS group.

NINDS scale	Δ*a*	Δ1/3	Δ*t*_1_ (ms)	Δ*t*_2_ (ms)	*T*_s_ (sec)	*V*_max_
0+	3.45 ± 1.93	0.82 ± 0.3	108 ± 71	1.78 ± 0.244	0.89 ± 0.318	2.73 ± 1.96
1+	24.52 ± 8.4	0.144 ± 0.12	354 ± 68	1.57 ± 0.164	1.97 ± 0.766	10.34 ± 5.06
2+	59.57 ± 12.41	0.156 ± 0.16	414 ± 64	1.73 ± 0.173	2.41 ± 0.785	26.97 ± 9.66
3+	93.83 ± 18.39	0.135 ± 0.16	440 ± 52	1.79 ± 0.222	2.53 ± 0.773	38.71 ± 9.53

**Table 2:** Classification accuracy for different feature combinations, showing the LOO CV testing performance.

	Naive Bayes (%)	Tree BAGGER (%)	KNN (%)	SVM (%)
Δ*a*, *V*_max_	89.62	82.07	86.79	67.92
Δ*a*, *V*_max_ (with PCA)	88.64	83.96	86.79	66.98
Δ*a*, *V*_max_, Δ1/3	84.9	86.79	83.96	69.81
Δ*a*, *T*_s_	86.79	84.9	35.84	71.69
Δ1/3, Δ*t*_1_, Δ*t*_2_	40.56	53.77	53.77	34.9
Δ1/3, Δ*t*_1_, Δ*t*_2_ (with PCA)	57.54	55.66	52.86	40.56

## Data Availability

The data used to support the findings of this study are available from the corresponding author upon request.
