# Corrigendum to “Classification and Assessment of the Patelar Reflex Response through Biomechanical Measures”

**DOI:** 10.1155/johe/9781582

**Published:** 2025-07-02

Y. Salazar-Muñoz, G. A. López-Pérez, B. E. García-Caballero, R. Muñoz-Rios, L. A. Ruano-Calderón, and L. Trujillo, “Classification and Assessment of the Patelar Reflex Response Through Biomechanical Measures,” *Journal of Healthcare Engineering* 2019, no. 1 (2019): 1–7, https://doi.org/10.1155/2019/1614963.

In the article titled “Classification and Assessment of the Patelar Reflex Response through Biomechanical Measures,” there was a spelling error in the title, where the term “Patellar” has been misspelt as “Patelar”. The correct title is shown below:

“Classification and Assessment of the Patellar Reflex Response through Biomechanical Measures”.

We apologize for this error.

